# MARCH5 Promotes Cardiac Hypertrophy by Regulating Akt/mTOR/Gsk‐3β/GATA4 Signalling Pathway

**DOI:** 10.1111/jcmm.70735

**Published:** 2025-08-03

**Authors:** Guoyong Li, Fengming Wu, Fan Lei, Jialiang Zhang, Yanbiao Liao

**Affiliations:** ^1^ Department of Cardiology, West China Hospital Sichuan University Chengdu China; ^2^ Laboratory of Cardiac Structure and Function, Institute of Cardiovascular Diseases, West China Hospital Sichuan University Chengdu China; ^3^ Cardiac Structure and Function Research Key Laboratory of Sichuan Province, West China Hospital Sichuan University Chengdu China

**Keywords:** cardiac hypertrophy, E3 ubiquitin ligase, heart failure, MARCH5, protein kinase B, ubiquitin‐proteasome system

## Abstract

This study aims to elucidate the role of MARCH5 in cardiac hypertrophy, thereby providing a theoretical foundation for novel therapeutic strategies for cardiac hypertrophy and heart failure. The expression of MARCH5 in cardiac hypertrophy models was assessed using immunohistochemistry, western blot (WB) and RT‐qPCR. Gain‐ and loss‐of‐function experiments of MARCH5 in cardiac hypertrophy were conducted both in vitro and in vivo. WB, RT‐qPCR, co‐immunoprecipitation (CoIP), immunohistochemistry and immunofluorescence were performed to investigate the molecular mechanisms of MARCH5. MARCH5 expression was upregulated in hypertrophied myocardium. Ang II stimulation resulted in increased expression of MYH7, BNP and cardiomyocyte area. These effects were aggravated by MARCH5 overexpression but antagonised by MARCH5 knockdown. MARCH5 heterozygous (MARCH5^+/−^) mice subjected to transverse aortic constriction (TAC) demonstrated alleviation of cardiac hypertrophy and improvement in cardiac function, whereas MARCH5 overexpression aggravated hypertrophy and cardiac dysfunction. Mechanistic studies indicated that MARCH5 directly interacted with Akt, enhancing the phosphorylation of Akt, mTOR and Gsk3β, thereby increasing GATA4 expression and aggravating cardiac hypertrophy. Our findings suggest that MARCH5 participates in the pathological cardiac hypertrophy by regulating the Akt/mTOR/Gsk‐3β/GATA4 pathway, positioning it as a promising therapeutic target for cardiac hypertrophy.

**Abbreviations**
AAV‐9: adeno‐associated virus serotype 9
CoIP: co‐immunoprecipitation
CVF: collagen volume fraction
DAPI: diamidinyl phenyl indole
DMEM: dulbecco's modified eagle medium
EF: ejection fraction
eNOS: endothelial nitric oxide synthase
FS: fraction shortening
GATA4: GATA Binding Protein 4
GFP: green fluorescent protein
Gsk‐3β: glycogen synthase kinase‐3 beta
HW/BW: heart weight/body weight
HW/TL: heart weight/tibia length
KO: knock out
LVAWd: diastolic left ventricular anterior wall thickness
MARCH5: membrane‐associated RING‐CH protein V
MOI: multiplicity of infection
mTOR: mammalian target of rapamycin
NRCF: neonatal rat cardiac fibroblasts
NRCM: neonatal rat cardiac myocytes
PCR: polymerase chain reaction
PKB/Akt: protein kinase B
PMSF: phenylmethanesulfonyl fluoride
RT‐qPCR: real‐time quantitative polymerase chain reaction
siRNA: small interfering ribonucleic acid
TAC: transverse aortic constriction
WB: western blot
WT: wild type

## 1 Introduction

Between 1990 and 2019, the global cases of cardiovascular disease increased from 271 million to 523 million, a trend that is primarily attributed to the aging population and advances in diagnostic and therapeutic technologies [1]. Cardiac hypertrophy is an adaptive response to both physiological and pathologic stimuli. However, prolonged pathological stress may lead to maladaptation, ultimately progressing to heart failure [2]. Research suggests that inhibiting myocardial hypertrophy under prolonged stress may help preserve cardiac function and delay the progression of heart failure [2, 3]. Understanding the mechanisms of pathological cardiac hypertrophy could provide novel therapeutic strategies for the treatment of both hypertrophy and heart failure.

The ubiquitin‐proteasome system (UPS) is crucial for maintaining protein homeostasis by facilitating protein degradation, which affects processes such as the cell cycle, cellular growth and differentiation [4, 5]. Ubiquitination is a multistep enzymatic process, including ubiquitin‐activating enzymes (E1), ubiquitin‐conjugating enzymes (E2) and ubiquitin ligases (E3). E3 ligases are particularly critical in determining the specificity of ubiquitination by selecting target substrates [6]. E3 ubiquitin ligases can be categorised into three primary families: the homologous to E6AP C‐terminus (HECT), the RBR E3s (with RING between RING structural domains) and the RING finger family [7].

MARCH5 (also known as MITOL) belongs to a subfamily of RING‐type E3 ubiquitin ligases, located in the mitochondrial outer membrane. Pressure overload‐induced cardiac hypertrophy is associated with significantly increased protein synthesis, where the rate of protein synthesis exceeds degradation, accompanied by an elevated expression of ubiquitin, E2 and E3 ligases [8, 9]. In addition, several E3 ubiquitin ligases including MuRF1 [10], Atrogin‐1 [11], Carboxyl terminus of HSP70‐interacting protein (CHIP) [12] and Tumour Necrosis Factor Receptor‐Related Factor 6 (TRAF6) [13], have been reported to participate in the pathological cardiac hypertrophy. However, the role of MARCH5 in pathological cardiac hypertrophy remains unclear and requires further investigation.

Many pro‐hypertrophic signalling pathways are regulated by ubiquitination, including the Wnt/β‐catenin, nuclear factors of activated T cells (NFAT), Janus kinase (JAK) and signal transducer and activator of transcription (STAT) pathways [14, 15, 16]. Previous studies have demonstrated that Akt activation is involved in pathological myocardial hypertrophy [17, 18, 19, 20]. mTOR and Gsk‐3β, as downstream molecules of Akt, are involved in mediating pathological cardiac hypertrophy [21]. Notably, GSK‐3β functions as a negative regulator of cardiac hypertrophy. GSK‐3β regulates the activity of proliferation regulators (GATA, cyclin D1 and c‐Myc) through phosphorylation, thereby influencing cardiomyocyte proliferation and cardiac hypertrophy [22, 23]. GATA4, as a transcriptional regulator, modulates the expression of a broad spectrum of cardiac genes, including those associated with cardiac hypertrophy, such as ANP, BNP and MHC [24, 25]. Furthermore, our previous finding suggests that MARCH5 can regulate the Akt signalling pathway and influence endothelial cell function via Akt/eNOS [26]. Given the critical role of E3 ubiquitin ligases (including MARCH5) and the Akt pathway in cardiac hypertrophy, we hypothesise that MARCH5 may be involved in the development of cardiac hypertrophy by regulating the Akt pathway. Furthermore, the role of MARCH5‐Akt in cardiac hypertrophy remains unreported. Targeting the regulation of MARCH5 and Akt pathways, either independently or in combination, could offer a novel therapeutic strategy for the treatment of pathological cardiac hypertrophy and even cardiac insufficiency.

In this study, we provide the evidence that MARCH5 plays a crucial role in cardiomyocyte hypertrophy. Mechanistically, MARCH5 was found to physically interact with Akt, and its downregulation significantly attenuated Akt phosphorylation along with subsequent inhibition of the downstream mTOR/Gsk‐3β/GATA4 signalling cascade. These molecular alterations ultimately contributed to the amelioration of cardiac hypertrophy. Our study identifies MARCH5 as a novel regulatory component in myocardial hypertrophy through its modulation of the Akt/mTOR/Gsk‐3β/GATA4 signalling axis.

## 2 Materials and Methods

### 2.1 Reagents

Angiotensin II (Ang II) was obtained from Sigma‐Aldrich (USA). Small interfering RNA targeting MARCH5 and negative control were obtained from GenePharma Bio (Shanghai, China). Plasmids for the overexpression of Flag‐MARCH5 and HA‐Akt, as well as AAV9 vectors for MARCH5 expression and corresponding empty vectors, were obtained from Hanbio (Shanghai, China). Antibodies targeting MARCH5, β‐TUBULIN and α‐actinin were purchased from Abcam (Cambridge, UK). Antibodies targeting BNP, MYH7 and GATA4 were obtained from ABclonal Technology (Wuhan, China). Antibody targeting Flag was obtained from Sigma‐Aldrich (USA). Antibodies targeting Akt, p‐Akt, AMPK, p‐AMPK, Erk, p‐Erk, p38, p‐p38, mTOR, p‐mTOR, ubiquitin, Gsk‐3β and p‐Gsk‐3β were obtained from Cell Signalling Technology (MA, USA).

### 2.2 Experimental Animals and Animal Surgery

All animal procedures were approved by West China Hospital, Sichuan University. The procedures were in accordance with the National Institutes of Health Guidelines for the Care and Use of Laboratory Animals. Male C57BL/6 mice and SD rats were obtained from Dashuo Biotechnology Co. Ltd. (Chengdu, China). MARCH5 knockdown (MARCH5^−/+^) mice with a C57BL/6J background were purchased from Cyagen Biosciences. The MARCH5^−/−^ mice could not be obtained because they die in utero. Heterozygous C57BL/6 mice were treated with global knockdown rather than cardiac‐specific knockdown of MARCH5. MARCH5^−/+^ mice were identified and screened through genotype analysis. PCR genotyping was performed with the following primers: MARCH5 common forward: TACCATTAAGAAAACGGAGTCGGC, MARCH5 mutant reverse: TCTAAAAGGTGACAGGAGAACTGG, MARCH5 wild type reverse: TCCATTAGTCCTTACCCAGCTTT, homozygotes: one band with 728 bp. Heterozygotes: two bands with 728 and 697 bp, wild type allele: one band with 697 bp. Male wild type (WT) mice were infected with adeno‐associated virus serotype 9 (AAV9) encoding MARCH5 and EGFP Flag (AAV9‐EGFP‐MARCH5‐Flag) to express MARCH5 in C57BL/6 mice.

Male mice aged 8–10 weeks were randomly assigned to the TAC and sham groups. The surgical procedure was primarily based on the literature [27]. After exposing the aortic arch, a 5–0 silk suture was carefully threaded around the aortic arch to ligate the blood vessels, thereby inducing a deliberate constriction of the aortic arch. Animals in the sham group underwent the same procedures as in the surgical group, but without ligation of the aortic arch.

### 2.3 Echocardiographic Assessment

Mice were anaesthetised with 1.5%–2% isoflurane via inhalation and subsequently positioned in the supine position on a plate. Ultrasound imaging was performed using the Small Animal High Frequency Ultrasound Imaging System (VEVO 3100, Canada) with a 40‐MHz (MX550) probe. Left ventricular ejection fraction (EF%), fractional shortening (FS%) and diastolic left ventricular anterior wall thickness (LVAWd) were measured.

### 2.4 Histomorphological Staining Analysis

Four weeks post‐TAC, the mice were weighed and recorded. The heart and tibial length were also measured. After removal, the heart was promptly fixed in 4% paraformaldehyde, subsequently dehydrated, embedded in paraffin and sectioned transversely at a thickness of 4 μm for haematoxylin‐eosin (HE) staining, Masson's trichrome staining, Sirius red staining, wheat germ agglutinin (WGA) staining and immunohistochemical staining.

### 2.5 Cardiomyocyte Culture and Treatment

Primary culture of neonatal rat cardiomyocytes (NRCM) was established following protocols described in previously published study [28]. Briefly, neonatal rat hearts were finely minced into pieces and digested with a mixture of trypsin and collagenase type II. To isolate cardiomyocytes from cardiac fibroblasts, a differential attachment culture method was used. Cardiomyocytes were cultured in DMEM‐H medium containing 10% fetal bovine serum and 5‐BrdU. The rat cardiomyocyte cell line (H9C2) and the 293 T cell line were kindly donated by the cell platform of Public Laboratory Center of West China Hospital, Sichuan University. Once the cells were confirmed to be ready for the experiment, they were starved for 12 h. The medium was then replaced with complete medium containing 1 μM Ang II, followed by a 24‐h incubation to induce the cardiomyocyte hypertrophy model.

### 2.6 Small Interfering RNA (siRNA), Plasmid and Adenovirus (adv) Transfection

Cells were transfected with siRNA targeting MARCH5 and negative control (siNC) when they reached 50%–70% confluence. The siRNA (5′‐3′GGUGCAGAGGAUCUACUAATT; 3′‐5'UUAGUAGAUCCUCUGCACCTT) was transfected into cells using Lipofectamine RNAiMAX (Life Technologies) in serum‐free Opti‐MEM (Gibco). The overexpression plasmid was transfected into cells using Lipofectamine 3000 (Life Technologies) in serum‐free Opti‐MEM. To overexpress MARCH5, a recombinant adenovirus encoding MARCH5 was constructed by Vigene Biology. Adenovirus transfection was performed following the manufacturer's protocol. Subsequent procedures were performed 24 h post‐transfection.

### 2.7 Measurement of Cellular Surface Area

Cardiomyocytes were seeded onto the coverslips and exposed to Ang II for 24 h. After treatment, cells were fixed with 4% paraformaldehyde. The cells were incubated with anti‐α‐actinin antibody at a dilution of 1:200, followed by a fluorescent secondary antibody (Alexa Fluor 647 goat anti‐mouse IgG, 1:200). Nuclear staining was performed using DAPI at a dilution of 1:1000. The relative cellular area was quantified and analysed using ImageJ software.

### 2.8 Western Blotting and Co‐Immunoprecipitation (Co‐IP) Analysis

Cardiac tissues or cells were lysed in RIPA buffer, and protein concentration was quantified using the BCA assay. The PVDF membranes were probed with primary antibodies, followed by incubation with appropriate secondary antibodies. Protein bands were visualised using an ECL system, and their signal intensities were quantified using Bio‐Rad analysis Image Lab software.

The IP lysates were incubated with the target‐specific antibodies at 4°C for 2 h, with rabbit normal IgG serving as a negative control. Subsequently, magnetic beads conjugated with protein A and G were added to the lysates and incubated overnight at 4°C to facilitate the immunoprecipitation of target proteins. The immunoprecipitated proteins were subsequently analysed by western blotting.

### 2.9 Real‐Time Quantitative Polymerase Chain Reaction (RT‐qPCR)

Total RNA was extracted from cardiac tissues or cells using TRIzol reagent (Invitrogen) according to the manufacturer's protocol. Reverse transcription was performed using the PrimeScript RT Reagent Kit (Takara) following the provided instructions. The RT‐PCR assay was subsequently performed on the CFX96 Real‐Time PCR Detection System (Bio‐Rad) using the EvaGreen Supermix Kit (Bio‐Rad). Primers specific to the target genes were synthesised by Sangon Biotech, with sequences listed in Table 1.

**TABLE 1:** Primers for RT‐PCR.

Genes	Forward sequence (5′‐3′)	Reverse sequence (5′‐3′)
r‐POSTN	TCGTTCGTGGCAGCACCTTC	TCGCCTTCAATGTGGATCTTCGT
r‐CTGF	CCCTGACCCAACTATGATGC	CCTTACTCCCTGGCTTTACG
r‐MARCH5	TGTGTGGAGCCCTTGTCTTTCC	TTGCGGTGTGCCTGACGTAA
r‐MYH7	GCAGACAGAGAATGGGGAGCTGTCC	TCGCAATCATGCCGGGCTGAC
r‐ANP	GGCCTTTTGGCTCCCAGGCC	CTAAGTGCCGCCCCCGCTTC
r‐BNP	GGAGGCGAGACAAGGGAGAACA	TGCTCTGGAGACTGGCTAGGAC
r‐RPS11	CCTGTCTGGTGTTGTGACGA	AGAGCATTGGCTACAGTCCC

### 2.10 Statistical Analysis

All data are presented as mean ± SD. Statistical comparisons between two groups were performed using the Student's *t*‐test, while one‐way ANOVA was used to compare the means between multiple groups. Statistical analysis was conducted using GraphPad Prism version 8.0.1. A two‐sided *p*‐value < 0.05 was considered statistically significant.

## 3 Results

### 3.1 MARCH5 Is Upregulated in Hypertrophic Mouse Heart Tissue and Cardiomyocytes

**FIGURE 1:**
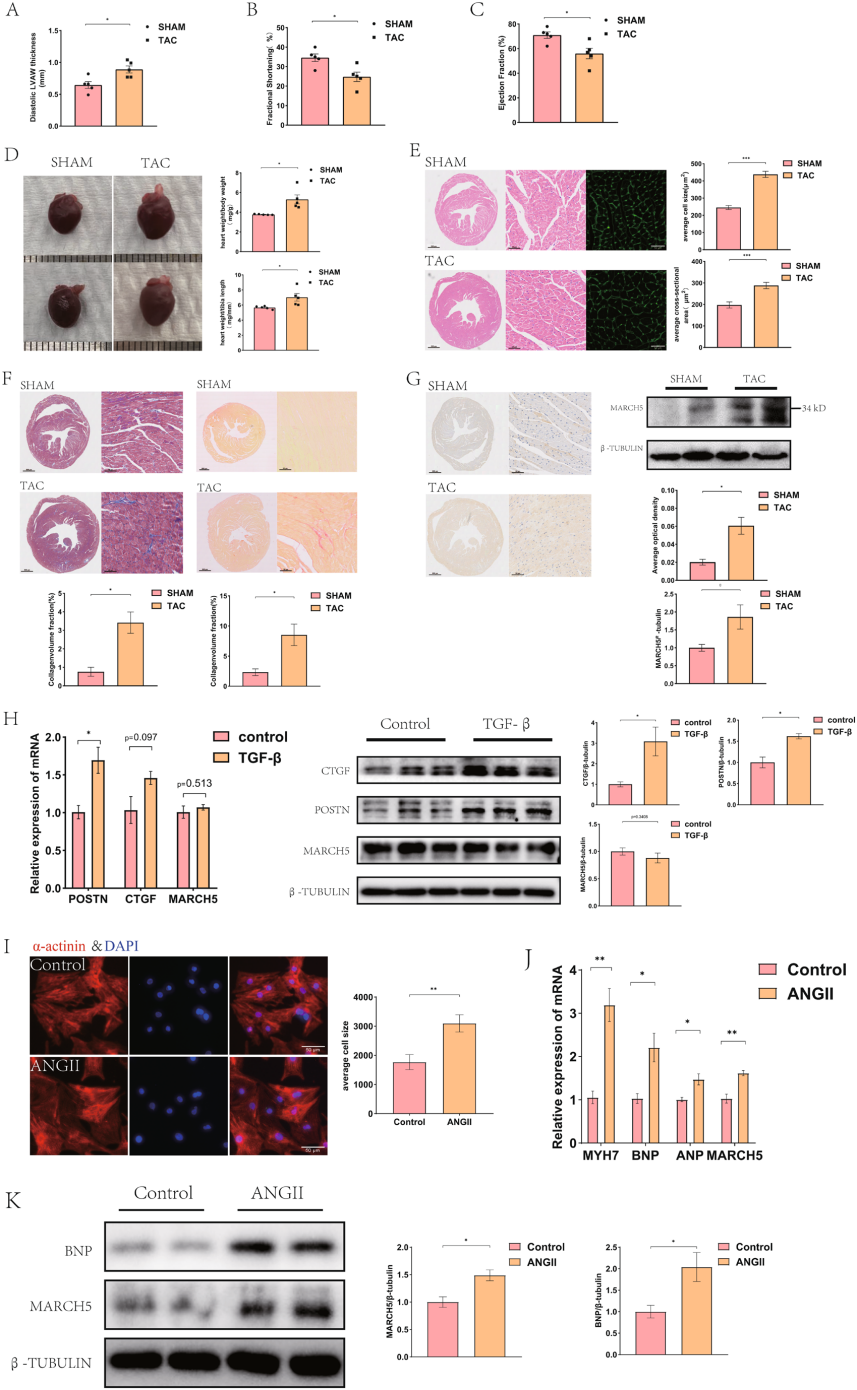
Expression of MARCH5 was upregulated in the myocardial hypertrophy model. (A–C) Cardiac functional tests showing diastolic left ventricular anterior wall thickness (LVAWd; panel A), left ventricular fraction shortening (FS%; panel B), and left ventricular ejection fraction (EF%; panel C) (*n* = 5). (D) Representative images of heart gross morphology and statistical results of heart weight/body weight (HW/BW) and heart weight/tibia length (HW/TL) ratios of mice in SHAM and TAC groups (*n* = 5). (E) Histological sections stained with HE and fluorophore‐conjugated WGA to measure cell size and myocyte cross‐sectional area in heart tissue (*n* = 5) [scale bar = 800 and 50 μm]. (F) Fibrosis in cardiac tissues was evaluated by Masson's Trichrome and Sirius red staining (*n* = 5) [scale bar = 800 and 50 μm]. (G) Representative immunohistochemical images stained for MARCH5 (MARCH5–brown, Nucleus‐blue) and data summary of average optical density and WB (*n* = 5) [scale bar = 800 and 50 μm]. (H) Treatment of NRCFs with TGF‐β. RT‐qPCR and WB to detect expression levels of POSTN, CTGF and MARCH5 in NRCFs (*n* = 3). (I) Representative immunofluorescence staining of NRCMs (α‐actinin‐red, Nucleus‐blue). Cell size was evaluated (*n* = 5) [scale bar = 50 μm]. (J) Treatment of NRCMs with Ang II. RT‐qPCR and WB were used to detect the expression of hypertrophic biomarkers and MARCH5 in NRCMs (*n* = 3). (K) NRCMs treated with Ang II. Western blotting analysis of MARCH5 and BNP expression (*n* = 3). *p < 0.05, **p < 0.01, ***p < 0.001.

To investigate the potential role of MARCH5 in pathological cardiac hypertrophy, we initially assessed MARCH5 expression levels in hypertrophic mouse hearts and cardiomyocytes. A mouse model of cardiac hypertrophy was established through transverse aortic constriction (TAC). Four weeks post‐TAC, echocardiography revealed increased left ventricular anterior wall thickness (LVAWd) (Figure 1A), accompanied by reductions in left ventricular ejection fraction (LVEF) and fractional shortening (FS) (Figure 1B,C and Figure S1A). Significant increases in the heart weight to body weight ratio (HW/BW) and tibia length (HW/TL) ratio were observed (Figure 1D). HE and WGA staining confirmed an increase in cardiomyocyte cross‐sectional area (Figure 1E). Masson's trichrome and Sirius red staining revealed an elevated collagen volume fraction (CVF), indicating the development of cardiac hypertrophy (Figure 1F). Immunohistochemistry and western blot were subsequently performed to assess MARCH5 alterations in cardiac hypertrophy (Figure 1G). Compared to the sham group, MARCH5 expression was significantly increased in the TAC group.

To identify the primary cellular target of MARCH5 in pathological cardiac hypertrophy, we isolated and cultured primary neonatal rat cardiac fibroblasts (NRCFs) and cardiomyocytes (NRCMs). TGF‐β stimulation of NRCFs increased mRNA and protein levels of POSTN and CTGF, confirming fibroblast activation, while MARCH5 expression remained unchanged (Figure 1H). The results indicated that Ang II (1 μM, 24 h) treatment of NRCMs could induce hypertrophy, resulting in elevated BNP, ANP and MYH7 expression and increased cell area (Figure 1I–K). Notably, MARCH5 levels also increased in hypertrophied cardiomyocytes, implicating its involvement in cardiac hypertrophy (Figure 1J,K). However, the expression of MARCH5 remained unchanged in Ang II‐treated NRCFs (Figure S2A). Based on these data, we focused on the effects of MARCH5 on cardiomyocytes.

### 3.2 Silencing of MARCH5 Alleviates Ang II Induced Cardiac Hypertrophy in NRCMs

**FIGURE 2:**
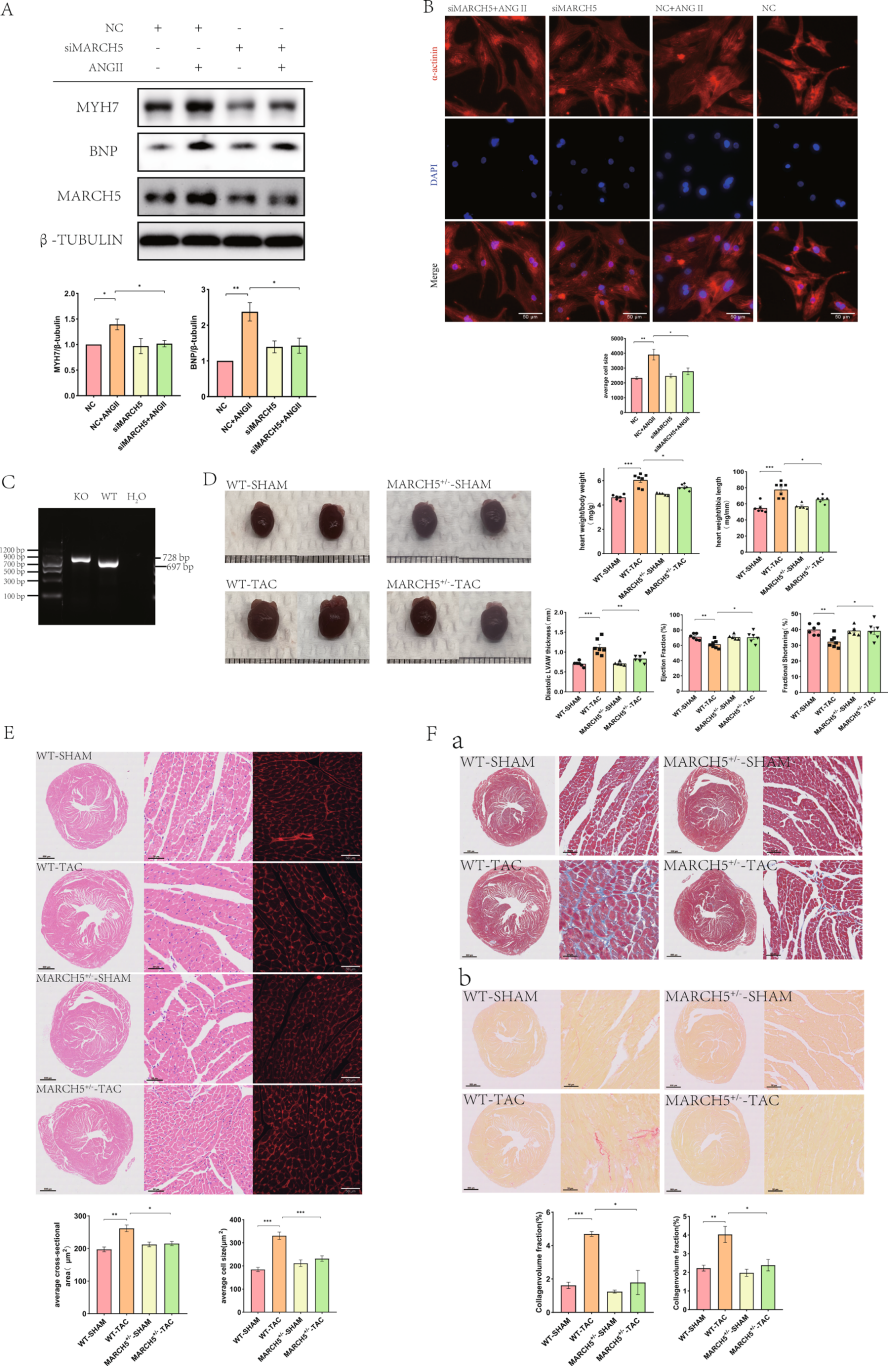
Knockdown of MARCH5 ameliorates cardiac hypertrophy both in vitro and in vivo. (A) WB detected the expression of MARCH5 and hypertrophic markers (MYH7 and BNP) in Ang II‐induced NRCMs transfected with SiMARCH5 (*n* = 4). (B) Representative immunofluorescence staining of NRCMs (α‐actinin‐red, Nucleus‐blue). Cell size was evaluated (*n* = 3) [scale bar = 50 μm]. (C) Representative image of genotyping assay for mouse tail. MARCH5 KO band (728 bp), MARCH5 WT band (697 bp), and negative control (pure water). (D) Representative images of heart gross morphology and statistical results of HW/BW and HW/TL ratios in mice (*n* = 5–7). Cardiac functional tests show LVAWd, FS%, and EF% (*n* = 5–7). (E) Histological sections stained with HE and fluorophore‐conjugated WGA were performed to measure the cross‐sectional area and size of mouse cardiomyocytes in each experimental group (*n* = 5–7) [scale bar = 800 and 50 μm]. (F) Fibrosis in cardiac tissues was assessed by Masson's Trichrome and Sirius red staining. Statistical analysis results of each experimental group (*n* = 5–7) [scale bar = 800 and 50 μm]. *p < 0.05, **p < 0.01, ***p < 0.001 .

To investigate the role of MARCH5 in pathological cardiac hypertrophy, NRCMs were transfected with MARCH5‐specific siRNA for 24 h prior to Ang II treatment. Post‐transfection, MARCH5 expression was downregulated in the siMARCH5 group compared to the siCtrl group (Figure 2A). Ang II treatment upregulated the hypertrophic markers BNP and MYH7; however, these effects were attenuated by MARCH5 knockdown (Figure 2A). Additionally, the cardiomyocyte area was also significantly increased by Ang II stimulation, which was mitigated by MARCH5 silencing (Figure 2B). These results suggest that silencing MARCH5 alleviates Ang II‐induced cardiac hypertrophy.

### 3.3 Knockdown of MARCH5 Attenuates TAC‐Induced Pathological Cardiac Hypertrophy in Mice

In vivo, the role of MARCH5 in TAC‐induced pathological cardiac hypertrophy was evaluated. Since homozygous MARCH5 knockout is lethal, heterozygous MARCH5 knockdown C57BL/6 mice were utilised, with WT littermates serving as controls. Genotyping of MARCH5 knockdown mice was performed using PCR amplification of DNA extracted from the mouse tail, following the manufacturer's protocol. The PCR results demonstrated a band at approximately 728 bp for the MARCH5 knockdown allele and a band at approximately 697 bp for the WT allele (Figure 2C). At baseline, no significant differences in cardiac phenotype, including heart size, HW/BW ratio, HW/TL ratio, cardiac function and ventricular wall thickness, were observed between MARCH5^+/−^ and WT mice (Figure 2D and Figure S1B). These findings suggest that partial knockdown of MARCH5 does not affect cardiac structure or function. However, following TAC, echocardiographic and anatomical analyses revealed that TAC‐induced cardiac abnormalities—such as increased ventricular wall thickness, elevated HW/BW and HW/TL ratios and reduced LVEF and LVFS—were more pronounced in WT mice compared to MARCH5^+/−^ mice (Figure 2D and Figure S1B). These findings indicate that MARCH5 knockdown provides protective effects on cardiac function following TAC stimulation.

Furthermore, TAC‐induced hypertrophic pathology was attenuated in MARCH5^+/−^ mice, as evidenced by HE and WGA staining, which demonstrated a comparatively milder increase in cardiomyocyte cross‐sectional area than in WT mice (Figure 2E). Additionally, fibrosis, assessed via Masson's trichrome and Sirius Red staining, revealed a lower collagen volume fraction in MARCH5^+/−^ mice post‐TAC compared to WT mice (Figure 2F). Notably, our findings revealed that neither TGF‐β nor Ang II had an impact on MARCH5 expression in vitro. Prior research has established that the activation of the renin–angiotensin–aldosterone system (RAAS) is pivotal in the progression of left ventricular hypertrophy (LVH), ultimately leading to the production of Ang II. Furthermore, Ang II is known to stimulate the activation of cardiac fibroblasts and cardiomyocytes [29, 30]. Consequently, we assessed the cardiac Ang II levels and observed a significant elevation in mice subjected to TAC. Intriguingly, knocking down MARCH5 resulted in a decrease in cardiac Ang II content (Figure S2B). Collectively, these results strongly suggest that MARCH5 silencing enhances resistance to pressure overload, implying that MARCH5 knockdown mitigates TAC‐induced ventricular remodelling.

### 3.4 Overexpression of MARCH5 Exacerbates Ang II‐Stimulated Hypertrophy in NRCMs

**FIGURE 3:**
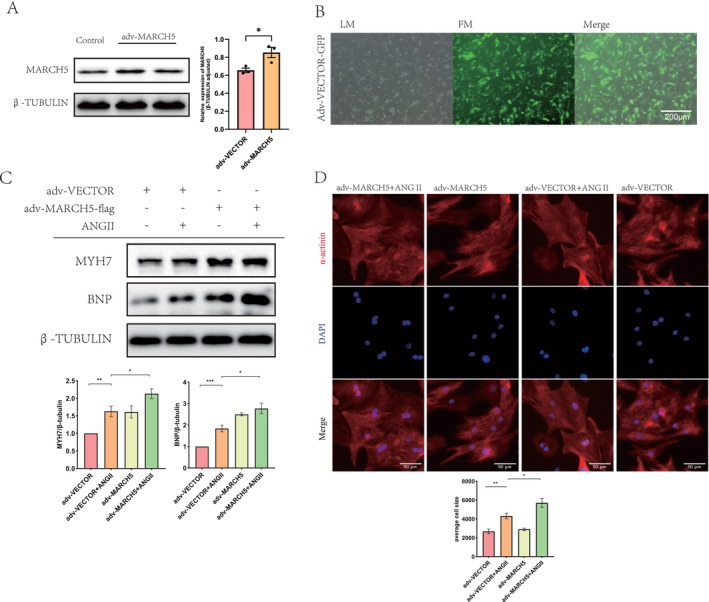
Overexpression of MARCH5 promotes Ang II‐induced cardiac hypertrophy. (A) WB detected the expression of MARCH5 transfected with adv‐MARCH5 and siMARCH5. (B) Representative immunofluorescence staining of NRCMs (adv‐VECTOR‐GFP, LM‐ Light microscopy, FM‐ Fluorescence microscope). (C) WB detected the expression of hypertrophy markers MYH7 and BNP in Ang II‐induced NRCMs transfected with adv‐MARCH5 (*n* = 4). (D) Immunofluorescence staining of Ang II‐induced NRCMs to assess cell size (α‐actinin‐red, Nucleus‐blue) [scale bar = 50 μm, (*n* = 3)]. *p < 0.05, **p < 0.01, ***p < 0.001.

To directly investigate the role of MARCH5 in cardiomyocyte enlargement, we constructed an adenovirus expressing MARCH5 (adv‐MARCH5) or adv‐VECTOR‐GFP to infect NRCMs. As demonstrated in Figure 3A,B, successful transfection of adv‐VECTOR‐GFP was confirmed. Compared to the corresponding controls, adv‐MARCH5 significantly upregulated the expression of hypertrophic markers, including MYH7 and BNP (Figure 3C). Consistently, MARCH5 overexpression led to an increase in cardiomyocyte size, as shown by α‐actinin immunofluorescence staining, indicating that exogenous MARCH5 overexpression exacerbates cardiomyocyte hypertrophy induced by Ang II stimulation (Figure 3D).

### 3.5 MARCH5 Overexpression Exacerbates TAC‐Induced Cardiac Hypertrophy in Mice

**FIGURE 4:**
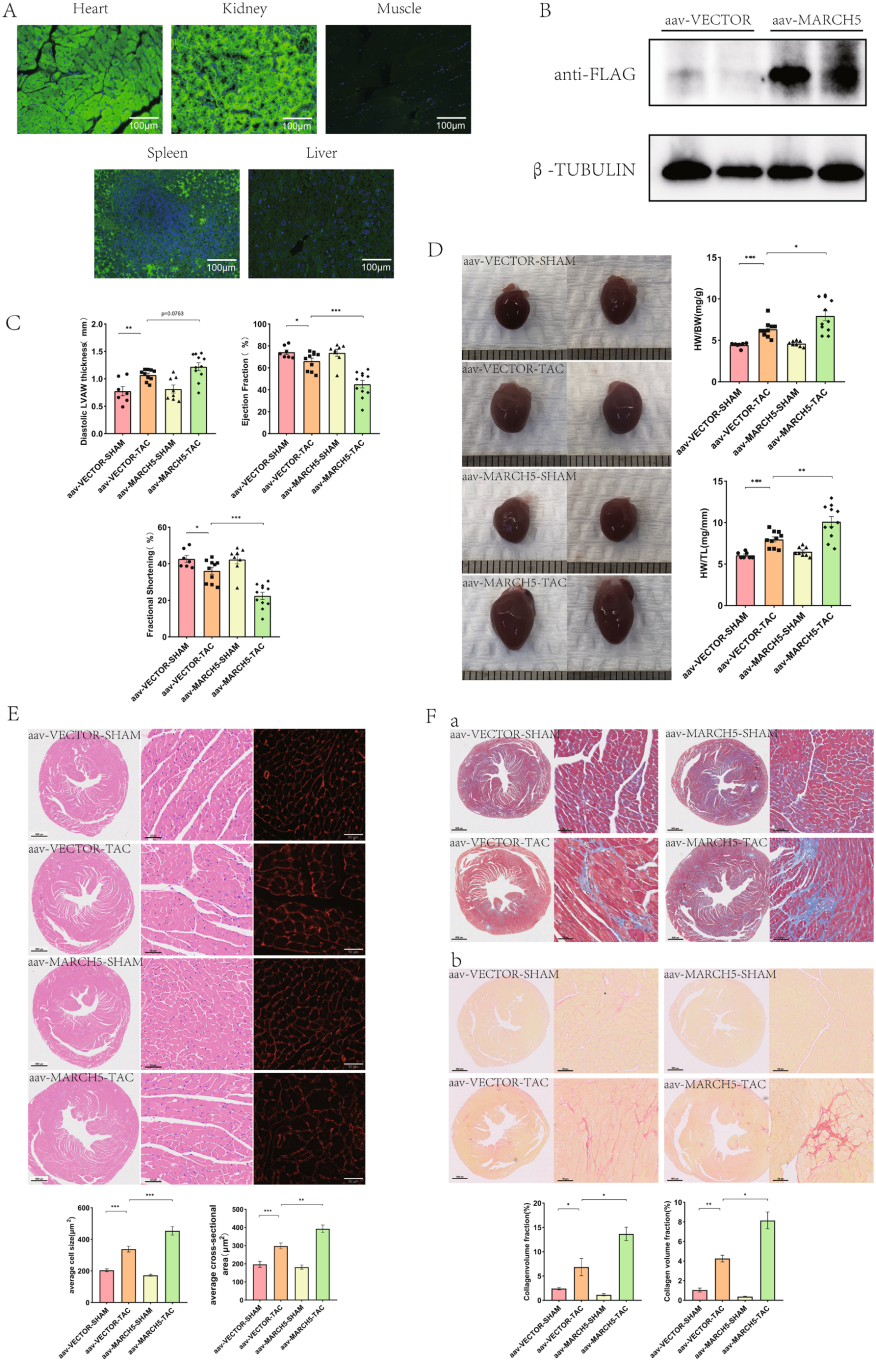
MARCH5 overexpression exacerbates pressure overload‐induced cardiac hypertrophy in mice. (A) Expression of the fluorescent tag GFP (green fluorescence) in various tissues of mice after injection of adeno‐associated virus (AAV9) via tail vein. Blue fluorescence represents DAPI. (B) Expression of exogenous MARCH5 protein (fusion with flag tag) in heart tissues detected by WB. (C) Statistical results of cardiac function indices in mice detected by echocardiography: EF%, FS%, and LVAWd (*n* = 7–11). (D) Representative heart gross morphology and statistical results of HW/BW and HW/TL ratios in mice (*n* = 7–11). (E) Histological sections stained with HE and fluorophore‐conjugated WGA were performed to measure the cross‐sectional area and size of mouse cardiomyocytes in each experimental group [scale bar = 800 and 50 μm, (*n* = 7–11)]. (F) Fibrosis in cardiac tissues was assessed by Masson's Trichrome and Sirius red staining. Statistical analysis results of each experimental group [scale bar = 800 and 50 μm, (*n* = 7–11)]. *p < 0.05, **p < 0.01, ***p < 0.001.

To further confirm whether in vivo overexpression of MARCH5 exacerbates myocardial hypertrophy, we utilised adeno‐associated virus (AAV9) vectors engineered to express MARCH5 (AAV9‐EGFP‐MARCH5‐flag) specifically in cardiomyocytes. Three days before‐TAC, mice were administered 100 μL of viral suspension containing 2 × 10^11^ viral particles via tail vein injection. 4 weeks later, the mice were humanely euthanised. Subsequently, the organs were excised and examined. Notably, heart tissues exhibited more intense green fluorescence than other organs (Figure 4A). Furthermore, robust detection of flag‐tagged protein in heart tissues confirmed successful overexpression of MARCH5 in mice injected with the AAV9‐EGFP‐MARCH5‐flag virus (Figure 4B), demonstrating that tail vein injection significantly elevated MARCH5 expression in cardiomyocytes.

Echocardiographic analysis revealed that MARCH5 overexpression markedly aggravated TAC‐induced cardiac hypertrophy, as demonstrated by an increase in LVAWd and a decrease in LVEF and LVFS compared to the AAV9‐vector‐TAC group, 4 weeks post‐TAC (Figure 4C and Figure S1A,B). Following echocardiographic assessment, the mice were humanely euthanised, and a meticulous dissection was performed to evaluate the organs, with particular attention to pathological changes. Consistent with in vitro findings, MARCH5 overexpression also significantly increased cardiomyocyte size, as evidenced by HE and WGA staining, as well as the HW/BW and HW/TL ratios, compared to the AAV9‐vector‐TAC group (Figure 4D,E). Histological analysis, including Masson's trichrome and Sirius Red staining, revealed that TAC‐induced interstitial fibrosis, assessed by collagen volume fraction, was further exacerbated by MARCH5 overexpression compared to the AAV9‐vector‐TAC group (Figure 4F). Moreover, cardiac Ang II levels were increased in TAC with MARCH5 overexpression (Figure S2B). In summary, these data demonstrate that MARCH5 overexpression exacerbates pressure overload‐induced cardiac hypertrophy and subsequent deterioration of cardiac function.

### 3.6 MARCH5 Interacts With Akt and Promotes Cardiac Hypertrophy via Regulating Phosphorylation of Akt

**FIGURE 5:**
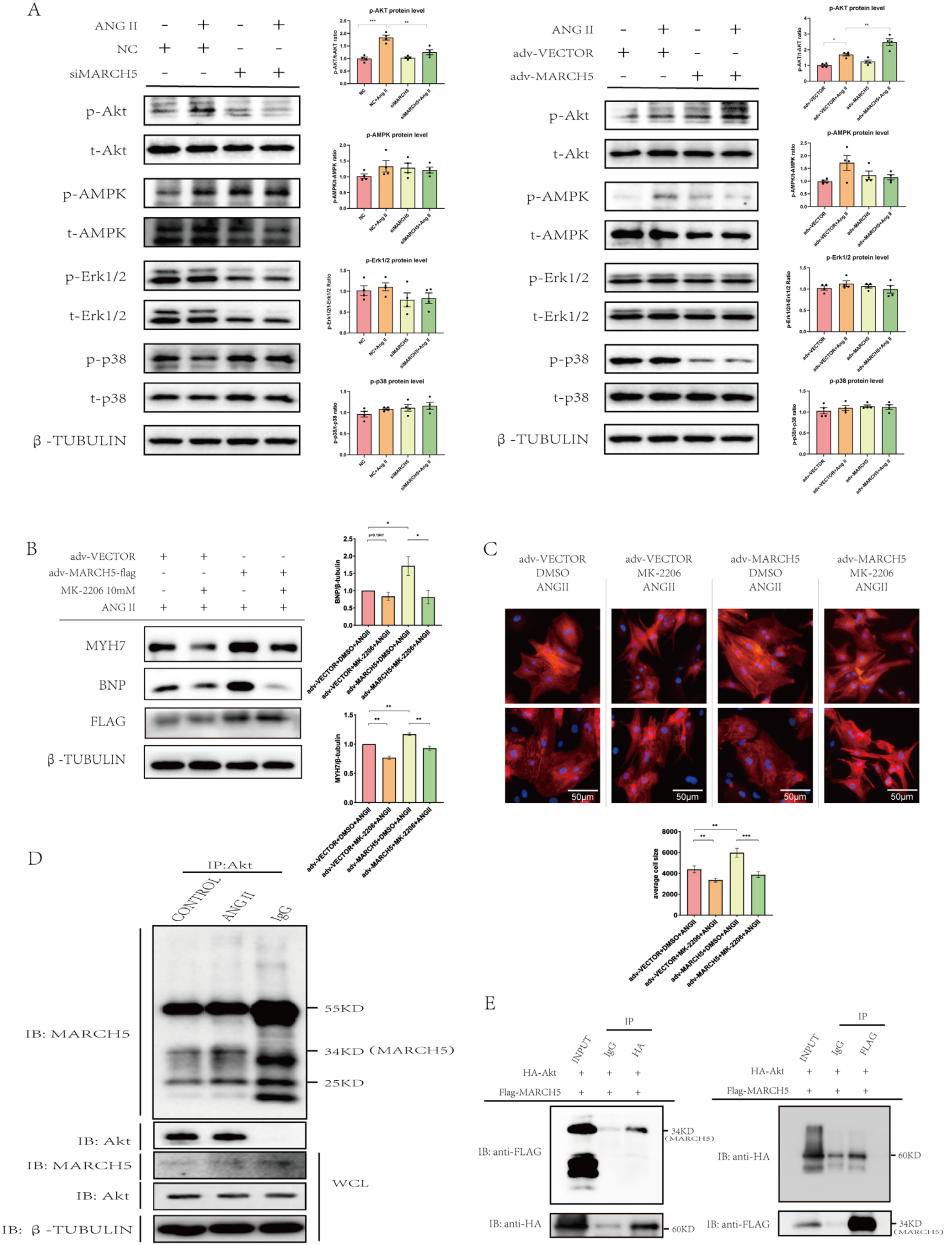
MARCH5 promotes cardiac hypertrophy through direct interaction with Akt and regulation of its phosphorylation. (A) MARCH5‐expressing vectors and siMARCH5‐transfected NRCMs were challenged with Ang II, and the phosphorylation levels of Akt, AMPK, Erk, and p38 MAPK were detected by WB. (B) MARCH5‐expressing vectors transfected NRCMs were pretreated with MK‐2206 for half an hour before Ang II stimulation, and WB detected the expression of hypertrophic markers (BNP and MYH7) (*n* = 3). (C) Representative immunofluorescence staining of NRCMs (α‐actinin‐red, Nucleus‐blue). Cell size was evaluated (*n* = 3). (D) H9C2 cells were treated as indicated in Panel D. Lysates from cells were immunoprecipitated with anti‐Akt, and normal rabbit IgG was used as a negative control. (E) The 293 T cell line was transfected with HA‐tagged Akt and FLAG‐tagged MARCH5 overexpression plasmid. Lysates from cells were immunoprecipitated with anti‐HA or anti‐Flag antibodies, and normal IgG antibody was used as a control. *p < 0.05, **p < 0.01, ***p < 0.001.

Protein kinases are essential for various cellular processes, as they catalyse protein phosphorylation. Dysregulation of these kinases is linked to numerous diseases, including cancer, cardiovascular disorders, neurodegenerative diseases, and inflammation [31]. Previous research has demonstrated that MARCH5 preserves the stemness of mouse embryonic stem cells by suppressing the Erk signalling pathway [32]. Building on this, we investigated the phosphorylation of key protein kinases, including Akt, AMPK, Erk and p38 MAPK, in Ang II‐induced cardiomyocyte hypertrophy. Western blot analysis revealed that Ang II stimulation significantly increased Akt and AMPK phosphorylation in NRCMs, whereas the Erk and p38 MAPK phosphorylation remained unchanged (Figure 5A). To determine whether MARCH5 modulates cardiomyocyte hypertrophy via the Akt or AMPK pathway, we employed siRNA‐mediated knockdown (siMARCH5) and advMARCH5‐mediated overexpression in NRCMs under Ang II stimulation. The results showed that MARCH5 inhibition significantly attenuated Ang II‐induced Akt phosphorylation, while MARCH5 overexpression markedly increased Akt phosphorylation compared to the control (Figure 5A). However, AMPK phosphorylation levels induced by Ang II stimulation remained unaffected by either inhibition or overexpression of MARCH5, compared to the control.

To confirm whether MARCH5 mediates cardiac hypertrophy via the Akt pathway, we utilised MK‐2206, a potent and specific inhibitor of all three Akt isoforms [33]. NRCMs were pretreated with MK‐2206 for 30 min before Ang II stimulation. The results showed that MK‐2206 effectively abrogated the pro‐hypertrophic effects of MARCH5 overexpression, as evidenced by reduced MYH7 and BNP expression and smaller cardiomyocyte area compared to the DMSO control (Figure 5B,C). Collectively, these data indicate that MARCH5 regulates cardiac hypertrophy through the Akt signalling pathway. To test whether MARCH5 directly interacts with Akt, we performed co‐immunoprecipitation assays in the H9C2 cardiomyocyte cell line. The cell lysates were subjected to immunoprecipitation with Akt antibody, followed by immunoblotting with the MARCH5 antibody. Co‐immunoprecipitation assays in H9C2 cells showed that MARCH5 interacts directly with Akt, as MARCH5 was precipitated by Akt antibodies with or without Ang II treatment (Figure 5D). Further validation using HA‐tagged Akt and Flag‐tagged MARCH5 in 293 T cells confirmed their direct interaction, demonstrating that MARCH5 and Akt physically associate even in the absence of Ang II stimulation (Figure 5E). Considering that MARCH5 is a member of the E3 ubiquitin ligase family, further study is need to investigate whether MARCH5 would have an effect on the ubiquitination level of Akt. . In conjunction with the findings of the previous experiments, we concluded that MARCH5 interacts directly with Akt and exerts a pro‐hypertrophic effect after modifying Akt through phosphorylation.

### 3.7 MARCH5 Mediates Cardiomyocyte Hypertrophy by Modulating the Akt/mTOR/Gsk‐3β/GATA4 Signalling Pathway

**FIGURE 6:**
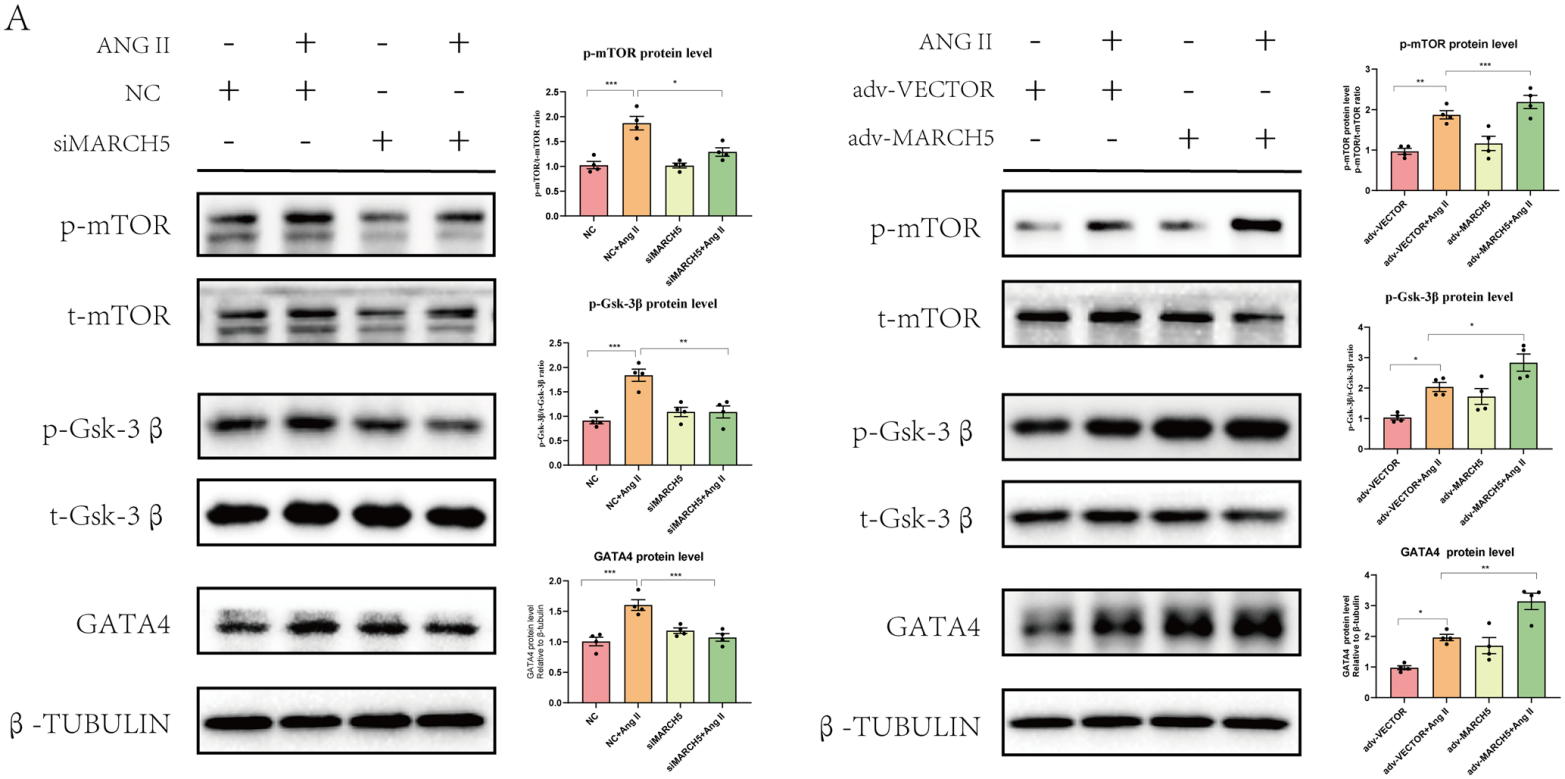
MARCH5 regulates Akt downstream signalling pathway. siMARCH5 and adv‐MARCH5 transfected NRCMs, the phosphorylation levels of Akt and its downstream signalling pathways, including p‐mTOR, p‐Gsk‐3β and GATA4 were detected by WB. *p < 0.05, **p < 0.01, ***p < 0.001.

Since MARCH5 directly interacts with Akt, we then examined the effect of MARCH5 on Akt downstream signalling. Western blot analyses showed that knockdown of MARCH5 led to a reduction in mTOR and Gsk‐3β phosphorylation levels, as well as GATA4 expression, in response to AngII stimulation, compared to the control group (Figure 6A). Conversely, MARCH5 overexpression resulted in increased mTOR and Gsk‐3β phosphorylation levels, as well as elevated GATA4 expression (Figure 6B). Prior research indicates that GATA4, a nuclear transcription factor, facilitates the expression of hypertrophic genes such as MYH7 and BNP, which ultimately contribute to myocardial hypertrophy [34, 35]. These results suggest that MARCH5 plays a crucial role in promoting cardiac hypertrophy through modulating the Akt/mTOR/Gsk‐3β/GATA4 signalling pathway.

## 4 Discussion

This study aimed to investigate the role of MARCH5, a member of the RING‐type E3 ubiquitin ligase family in pathological hypertrophy, revealing that MARCH5 is upregulated in cardiomyocytes in response to Ang II stimulation. MARCH5 knockdown attenuated cardiomyocyte hypertrophy, whereas its overexpression exacerbated hypertrophy under Ang II stimulation. These findings were further validated in MARCH5^+/−^ knockdown and AAV9‐MARCH5 overexpressed mice subjected to TAC‐induced pressure overload. Notably, we found that TGF‐β did not influence MARCH5 expression in vitro. However, in the TAC‐induced mouse hypertrophy model, both upregulation and downregulation of MARCH5 modulated the extent of cardiac fibrosis. TAC treatment induces a stress response in mice, secondary to the production of renin and angiotensin‐converting enzyme (ACE). Previous studies have demonstrated that activation of RAAS plays a critical role in the progression of LVH, leading to the generation of angiotensin II [29, 30], whereas Ang II induces cardiac fibroblast activation. In parallel, our results demonstrated that knockdown of MARCH5 reduced Ang II concentration in cardiac tissues, while overexpression of MARCH5 had the opposite effect. These results may partially demonstrate that MARCH5 knockdown or overexpression regulated TAC‐induced cardiac fibrosis even though TGF‐β did not affect MARCH5 expression in vitro. Mechanistically, MARCH5 was shown to interact with Akt, thereby activating the Akt/mTOR/Gsk‐3β/GATA4 signalling pathway. These results provide the first evidence that MARCH5 promotes cardiac hypertrophy via the Akt/mTOR/Gsk‐3β/GATA4 signalling pathway.

The UPS is essential for maintaining protein homeostasis, with its dysregulation implicated in cardiovascular diseases such as cardiac hypertrophy, heart failure and atherosclerosis [36]. E3 ligases, which determine substrate specificity in ubiquitination, are increasingly recognised for their role in pathological cardiac hypertrophy, with ligases such as TRAF6, TRIM16 and WWP1 being identified as key contributors [37, 38, 39]. MARCH5, a member of the E3 ligase family expressed in various organs, including the heart, has been shown to regulate mitochondrial dynamics by ubiquitylating Fis1, Mfn1, and Mfn2 [40]. Recent studies have highlighted MARCH5's involvement in cardiovascular diseases. For instance, Kitakata et al. found that MARCH5 knockdown enhances cardiomyocyte susceptibility to doxorubicin toxicity [41]. Zhang et al. demonstrated that silencing MARCH5 alleviates high glucose‐induced mitochondrial dysfunction and apoptosis in cardiomyocytes [42]. Additionally, another research revealed that MARCH5 overexpression mitigates cardiac dysfunction after myocardial infarction in mice [43]. Our previous work also identified that MARCH5 regulates endothelial cell function through the eNOS pathway against ischaemic/hypoxia injury [26]. However, the role of MARCH5 in pathological hypertrophy remains unclear. Our results demonstrate that MARCH5 expression is elevated in a mouse model of pressure overload‐induced cardiac hypertrophy and in Ang II‐induced NRCMs hypertrophy. MARCH5 knockdown alleviated pathological hypertrophy, while its overexpression exacerbated pathological hypertrophy both in vivo and in vitro. Therefore, MARCH5 plays a critical role in pressure overload‐induced cardiac hypertrophy.

Pathological cardiac hypertrophy is a complex process involving the activation of various intracellular signalling pathways. Cardiomyocytes respond to hypertrophic stimuli through a range of signalling cascades, leading to distinct phenotypes such as increased protein synthesis, altered metabolism, apoptosis, fibrosis, contractility and inflammatory responses [2, 44]. Among these, the protein kinase B (PKB, also known as Akt) pathway plays a pivotal role [17, 45]. Activation of the PI3K‐Akt and Akt/eNOS pathways inhibits the pathophysiological process of cardiac hypertrophy [46, 47]. In contrast, inhibition of the Akt‐FoxO1 and Akt/NF‐κB pathways exerts an anti‐hypertrophic effect [19, 48]. Our results demonstrate that MARCH5 promotes cardiomyocyte hypertrophy through the Akt pathway, and inhibition of Akt by MK2206 partially reverses MARCH5‐mediated hypertrophy, suggesting that targeting the Akt pathway could be a therapeutic strategy for pathological hypertrophy. Previous studies have shown that post‐translational modifications, such as methylation and ubiquitination, can affect Akt translocation and phosphorylation [31, 49, 50]. For instance, TRAF6‐mediated ubiquitination of Akt promotes its membrane translocation and activation, enhancing downstream signalling [50]. Our study demonstrated that MARCH5 binds directly to Akt, affecting its activation and downstream effectors, though the precise mode of interaction remains unclear and requires further investigation.

mTOR, a key serine/threonine protein kinase in the PI3K‐associated kinase family, regulates cellular growth, metabolism, protein synthesis and degradation [2, 51]. Activation of mTORC1 has been shown to drive pathological hypertrophy, whereas its inhibition reduces hypertrophy and improves cardiac function [52, 53]. In our study, we found that activation of mTOR was involved in Ang II‐stimulated cardiomyocyte hypertrophy, and changes in MARCH5 expression correlated with altered mTOR phosphorylation, suggesting that mTOR is involved in MARCH5‐mediated hypertrophy.

Gsk‐3β, a negative regulator of cardiac hypertrophy and a downstream target of Akt, is inhibited through Akt‐mediated phosphorylation [54, 55]. GSK‐3β mediates the cardiac hypertrophy‐protective effects of a low‐carbohydrate diet, and its activation is also involved in the anti‐hypertrophic effects of quercetin [56, 57]. Active Gsk‐3β suppresses hypertrophic gene transcription via factors such as GATA4, β‐catenin, c‐Myc and NFAT [58]. Relevant studies have demonstrated that Gsk‐3β plays an important role in several cardiovascular diseases, including cardiac hypertrophy and heart failure [59]. In particular, activated Gsk‐3β can drive GATA4 out of the nucleus via the nuclear transporter protein Crm1, reducing GATA4‐mediated ANP expression. However, it remains unclear whether Gsk‐3β directly affects GATA4 [60]. Our results showed that changes in MARCH5 expression also correlated with altered Gsk‐3β phosphorylation and GATA4 expression. These findings suggested that the MARCH5‐mediated Akt/mTOR pathway inactivated Gsk‐3β, reducing its inhibition of GATA4, which subsequently drove hypertrophic gene expression.

While our results were validated in cellular and animal models, human myocardial tissue samples for MARCH5 analysis were unavailable. Furthermore, although we identified a direct interaction between MARCH5 and Akt, the specific binding site and interaction mode remain unclear. Additionally, MARCH5, a mitochondrial outer membrane protein, may influence mitochondrial dynamics—an area that warrants future investigation.

## 5 Conclusion

In conclusion, we demonstrate that MARCH5 promotes cardiac hypertrophy through the Akt/mTOR/Gsk‐3β/GATA4 pathway. Inhibition of MARCH5 could provide potential therapeutic strategies for pathological cardiac hypertrophy.

## Author Contributions

**Guoyong Li:** conceptualization (lead), investigation (lead), methodology (equal), project administration (equal), writing – original draft (equal). **Fengming Wu:** formal analysis (equal), methodology (equal), visualization (equal), writing – original draft (lead). **Fan Lei:** formal analysis (equal), visualization (equal), writing – original draft (equal). **Jialiang Zhang:** formal analysis (equal), funding acquisition (equal), methodology (equal), writing – review and editing (equal). **Yanbiao Liao:** funding acquisition (lead), project administration (equal), supervision (lead), writing – review and editing (lead).

## Conflicts of Interest

The authors declare no conflicts of interest.

## Supporting information


Figure S1.

## Data Availability

The data that support the findings of this study are available from the corresponding author upon reasonable request.
