# Supplementary material for: MARCH5 Promotes Cardiac Hypertrophy by Regulating Akt/mTOR/Gsk‐3β/GATA4 Signalling Pathway
Source: J Cell Mol Med. 2025 Aug 3;29(15):e70735. doi: 10.1111/jcmm.70735 (PMC12318481; doi:10.1111/jcmm.70735)
Supplement: Supplementary file 1 — Figure S1. [file JCMM-29-e70735-s001.zip › Supplementary Materials.docx]

**MARCH5 promotes cardiac hypertrophy by regulating Akt/mTOR/Gsk-3β/GATA4 signaling pathway**

**- SUPPLEMENTARY MATERIALS –**

Guoyong Li^1^, Fengming Wu^1^, Fan Lei^1^, Jialiang Zhang^1,2,3^, Yanbiao Liao^1^

^1^ Department of Cardiology, West China Hospital, Sichuan University, Chengdu, China

^2^ Laboratory of Cardiac Structure and Function, Institute of Cardiovascular Diseases, West China Hospital, Sichuan University, Chengdu, 610041, PR China.

^3^ Cardiac Structure and Function Research Key Laboratory of Sichuan Province, West China Hospital, Sichuan University，Chengdu, 610041, PR China.


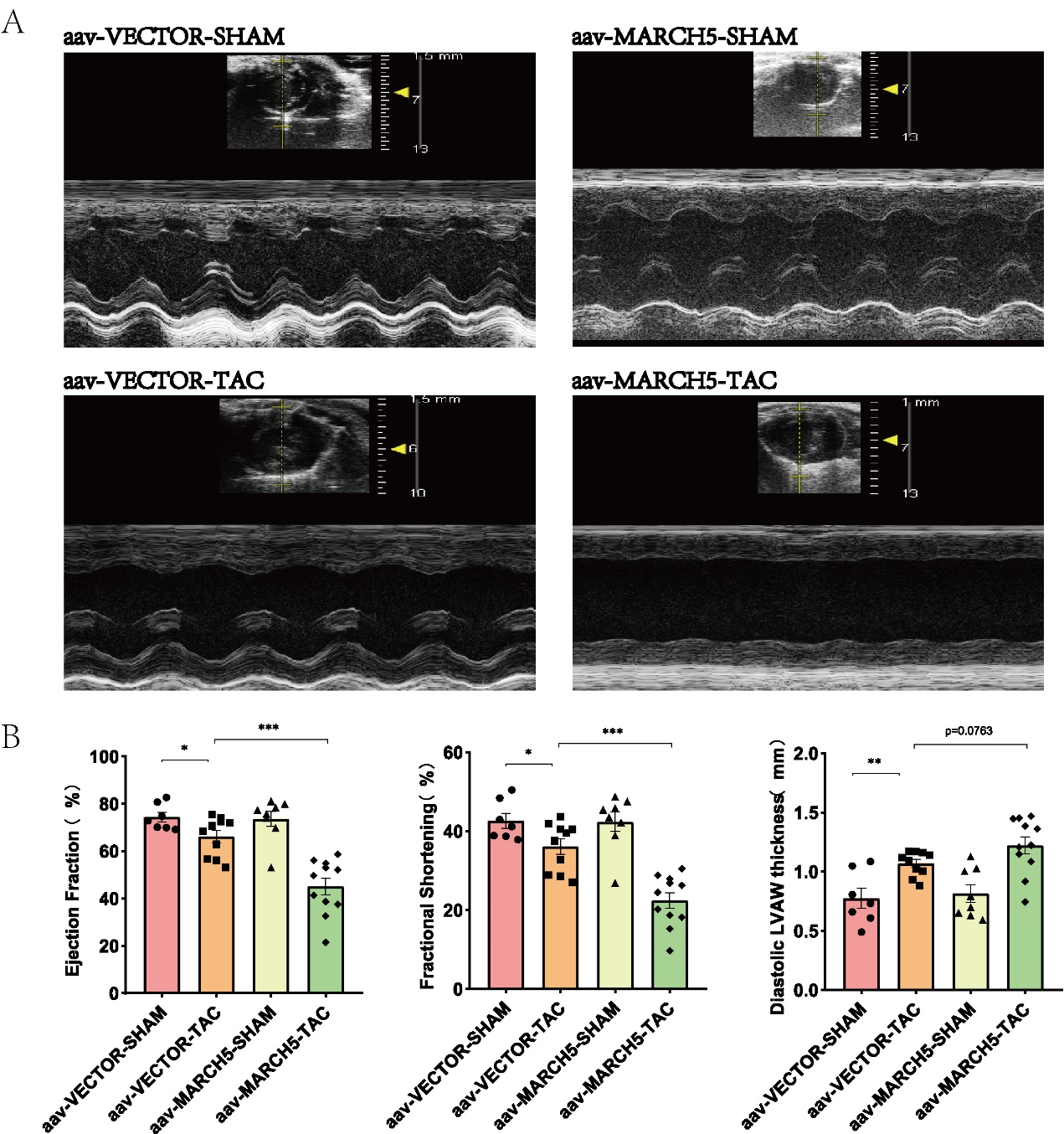


**Supplementary Figure S1**. Changes in cardiac ultrasound indices in mice after MARCH5 overexpression in vivo.

A. Echocardiographic cardiac function images of mice. B. Statistical results of cardiac function indices in mice detected by echocardiography: left ventricular ejection fraction (EF%), left ventricular fractional shortening (FS%), and diastolic left ventricular anterior wall thickness (LVAEd). *Indicates statistical differences between groups, *p<0.05, **p<0.01,***p<0.001, n=7-11.


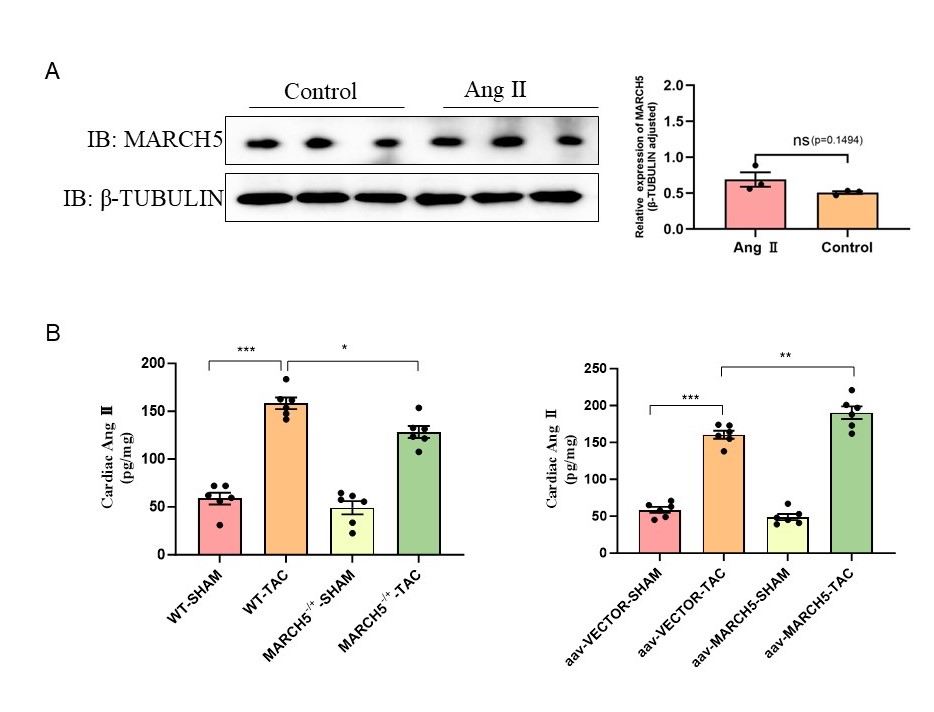


**Supplementary Figure S2**. Interrelationships between MARCH5 and Ang II.

A. MARCH5 expression was assessed in Ang II-treated NRCFs using WB (n=3). B. Cardiac Angiotensin II (Ang II) concentrations were measured in mice subjected to transverse aortic constriction (TAC) with or without the overexpression or inhibition of MARCH5 (n=6). *Indicates statistical differences between groups, *p<0.05, **p<0.01, ***p<0.001.


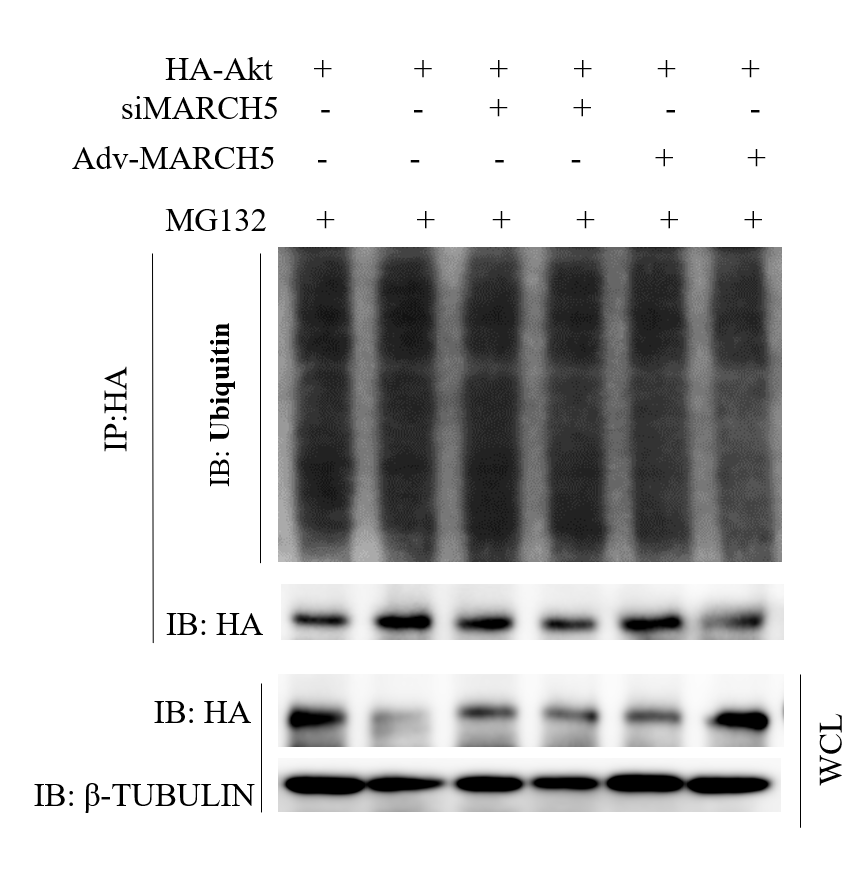


**Supplementary Figure S3.** MARCH5 does not affect Akt ubiquitination.

Immunoprecipitation was employed to investigate the impact of MARCH5 on Akt ubiquitination in response to adenovirus-mediated MARCH5 overexpression (adv-MARCH5) and MARCH5 knockdown (siMARCH5).
