# Supplementary figures and images for: MARCH5 Promotes Cardiac Hypertrophy by Regulating Akt/mTOR/Gsk‐3β/GATA4 Signalling Pathway
Source: J Cell Mol Med. 2025 Aug 3;29(15):e70735. doi: 10.1111/jcmm.70735 (PMC12318481; doi:10.1111/jcmm.70735)

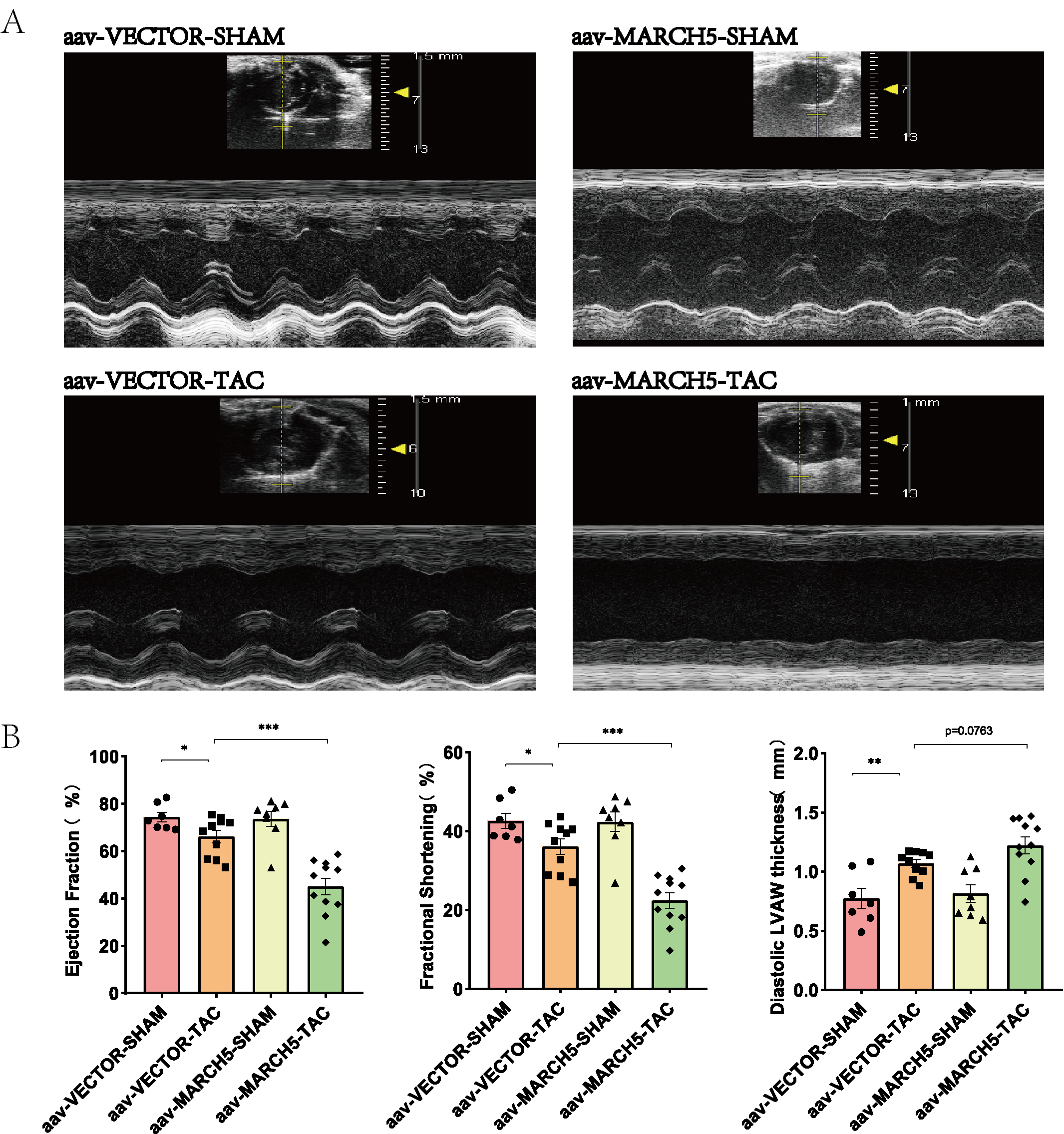

Supplement: Supplementary file 1 — Figure S1. [file JCMM-29-e70735-s001.zip › Figure S1.jpg]

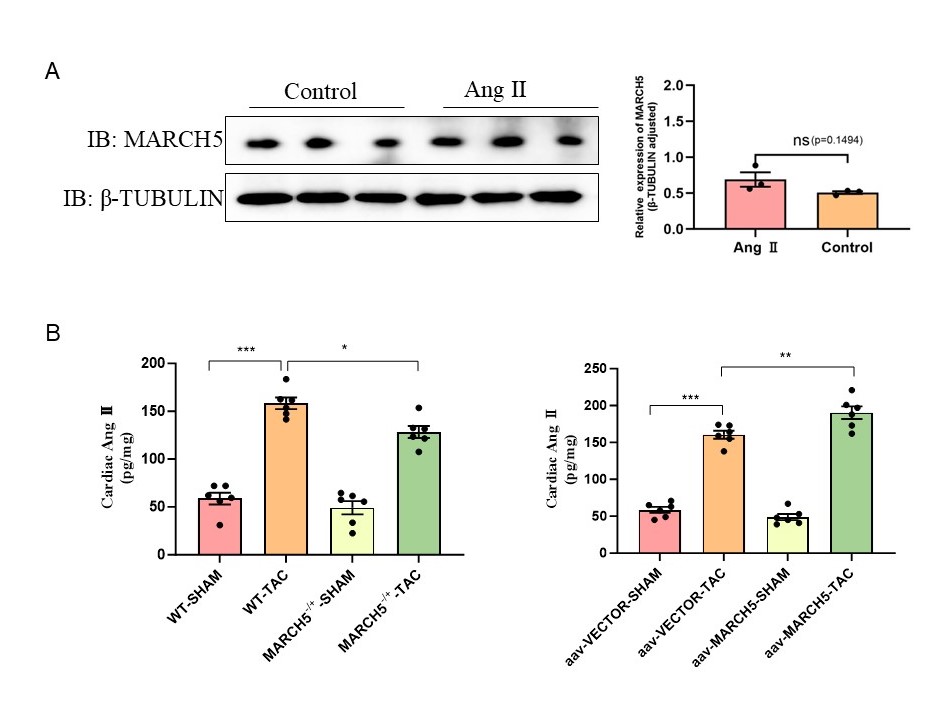

Supplement: Supplementary file 1 — Figure S1. [file JCMM-29-e70735-s001.zip › Figure S2.jpg]

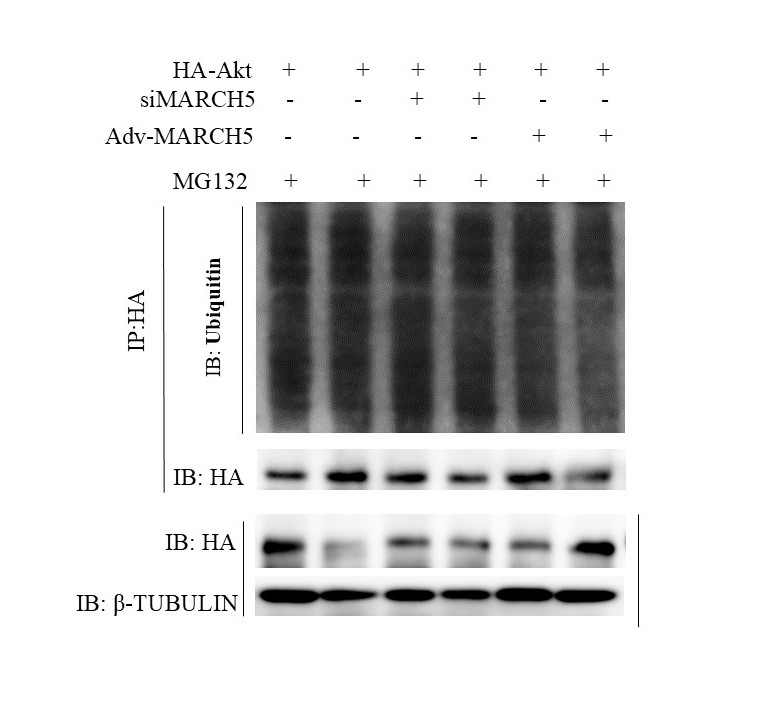

Supplement: Supplementary file 1 — Figure S1. [file JCMM-29-e70735-s001.zip › Figure S3.jpg]
